# ESRP2 constrains EMT plasticity associated with ZEB1 expression in bladder cancer

**DOI:** 10.3389/fonc.2026.1764850

**Published:** 2026-03-30

**Authors:** Karolina Bajdak-Rusinek, Karolina Jankowska, Vignesh Sundararajan, Łukasz Sieroń, Natalia Diak, Weronika Wójtowicz, Karolina L. Stępień, Agnieszka Fus-Kujawa, Ewa Gutmajster, Mateusz Wierzbinka, Kinga Zorychta

**Affiliations:** 1 Department of Molecular Biology, Medical University of Silesia, Katowice, Poland; 2 Students Scientific Society, Medical University of Silesia, Katowice, Poland; 3 Cancer Science Institute of Singapore, National University of Singapore, Singapore, Singapore; 4 Biotechnology Centre, Silesian University of Technology, Gliwice, Poland

**Keywords:** alternative splicing, bladder cancer, cancer stemness, EMT, ESRP2

## Abstract

**Introduction:**

Epithelial-to-mesenchymal transition (EMT)-driven phenotypic plasticity promotes bladder cancer (BC) progression and therapy resistance. While EMT has been primarily associated with transcriptional reprogramming, the contribution of post-transcriptional mechanisms, particularly alternative splicing regulation, remains insufficiently explored. This study aimed to investigate the clinical significance and mechanistic role of epithelial splicing regulatory protein 2 (ESRP2) in BC.

**Methods:**

Integrative analyses of publicly available transcriptomic datasets (TCGA and GEO) were performed to evaluate the prognostic value of ESRP2 and its association with epithelial and mesenchymal phenotypes in BC cell lines. Functional assays, including ESRP2 knockdown and overexpression, were conducted to assess its impact on EMT marker expression, cellular behavior, and stemness-related features such as clonogenicity, spheroid formation, and cell surface marker expression.

**Results:**

High ESRP2 expression correlated with improved patient survival and an epithelial-like phenotype in BC models. ESRP2 loss induced mesenchymal marker expression and increased cell motility, whereas ESRP2 overexpression restored epithelial morphology, reduced migration, and suppressed anchorage-independent growth. Flow cytometry revealed no significant changes in CD44 expression but showed a moderate increase in CD133+ cells following ESRP2 overexpression, suggesting a qualitative shift in stem-like subpopulations rather than a global suppression of cancer stemness.

**Discussion:**

These findings identify ESRP2 as a key post-transcriptional regulator that constrains EMT-associated transcriptional programs linked to ZEB1 expression, thereby stabilizing epithelial identity in bladder cancer. Targeting alternative splicing may represent a promising therapeutic strategy to limit tumor aggressiveness and overcome treatment resistance.

## 1 Introduction

Bladder cancer is a clinically and molecularly heterogeneous disease, with urothelial carcinoma being the most common histological subtype (1, 2). A critical feature underlying its progression, recurrence, and therapeutic resistance is phenotypic plasticity — the capacity of tumor cells to transition between epithelial and mesenchymal states through dynamic cellular reprogramming (3, 4). This epithelial-to-mesenchymal transition (EMT) promotes migratory and invasive behavior, acquisition of stem-like traits, and metastatic dissemination (5–7). EMT is driven by transcription factors such as ZEB1, SNAIL, and TWIST, which repress epithelial genes and activate mesenchymal transcriptional programs (8). Conversely, mesenchymal-to-epithelial transition (MET) is essential for metastatic colonization, enabling disseminated tumor cells to regain epithelial features and proliferative capacity (9).

While transcriptional control of EMT has been extensively characterized, the contribution of post-transcriptional regulation — particularly alternative splicing — to epithelial plasticity remains less well understood. Epithelial splicing regulatory proteins 1 and 2 (ESRP1 and ESRP2) are central mediators of epithelial-specific splicing programs (10, 11). Initially identified through their regulation of isoforms such as FGFR2 and CD44, ESRPs are now recognized as master regulators of epithelial identity (12), and their downregulation has been linked to EMT across multiple solid tumors (13–17). However, the specific role of ESRP2 in controlling phenotypic plasticity and stem-like features in bladder cancer remains poorly defined.

In this study, we identify ESRP2 as a clinically and mechanistically relevant regulator of cell-state identity in bladder cancer. Analyses of patient-derived transcriptomic datasets revealed that high ESRP2 expression correlates with favorable prognosis and an epithelial-like transcriptional profile. Using loss- and gain-of-function approaches, we demonstrate that ESRP2 constrains EMT-associated behavior, including migration, clonogenicity, and spheroid growth. Importantly, ESRP2 did not diminish the CD44^+^ stem-like compartment but increased the proportion of CD133^+^ epithelial progenitor-like cells, indicating a qualitative reprogramming of stemness rather than its depletion. Although ESRP1 shares partially overlapping functions, our results indicate that ESRP2 exerts a dominant and broader influence on epithelial identity and tumor cell plasticity.

We hypothesized that ESRP2 constrains EMT-associated programs linked to ZEB1 expression, thereby stabilizing epithelial identity and limiting invasive traits in bladder cancer.

Collectively, these findings establish ESRP2 as a post-transcriptional regulator that stabilizes epithelial differentiation states, highlighting its potential as both a prognostic biomarker and a therapeutic target in aggressive bladder cancer.

## 2 Materials and methods

### 2.1 Cell culture

Cell lines 5637, RT-4, T-24, and UM-UC-3 were purchased from American Type Culture Collection (ATCC). Cell lines were cultured in an appropriate growth medium supplemented with 10% fetal bovine serum (Gibco, Darmstadt, Germany, 10500064) at 37°C and 5% CO_2_. RT-4 and T-24 cell lines were cultured in McCoy’s 5A medium (ATCC, 30-2007), the 5637 line in RPMI-1640 (ATCC, 30-2001), and UM-UC-3 in EMEM medium (ATCC, 30-2003).

### 2.2 Knockdown and overexpression assay

Gene silencing was carried out by transfecting cells with small interfering RNAs (siRNAs). The 5637 and RT-4 cell lines were seeded in 12-well plates at a density of 5 × 10^4^ cells per well. After 24 hours, siRNAs purchased from Genomed (Poland) were introduced into the cells using Oligofectamine™ Transfection Reagent (Invitrogen), in accordance with the manufacturer’s protocol. The siRNA sequences used for knockdown were as follows:

siESRP1, 5’-CACAAUGACAGAGUAUUUA-3’;

siESRP2, 5’-GACUUAAUCCUCCUAGUUU-3’;

Control transfections were done with non-targeting siRNA (Ambion 4390847).

### 2.3 Overexpression construct preparation

The open reading frames (ORFs) encoding ESRP1 and ESRP2 amplified from cDNA synthetized of RNA from RT-4 cells (ATCC, USA). Amplification performed with half nested PCR with primer pairs for ESRP1; EN1R and E1F, whereas for ESRP2; NE2R and E2F. Amplification performed in 25 µL reaction with Perpetual OPTI taq Polymerase (EURx, Poland) with 5 µL of cDNA as template, annealing temperature was 55°C with 2.5 minutes of elongation. Subsequently, 2 µL of PCR reaction were taken as template for another PCR reaction with primers: for ESRP1: E1F and E1R, for ESRP2: E2F and E2R. Primers introduced overhand for Gibson cloning. Products were separated in 2% agarose gel, and purified by Gel Out kit (EurX, Poland).

pIRESneo3 plasmid (Takara Bio, USA) was cut with EcoRI (EurX, Poland), gel purified, and ESRP1 or ESRP2 cDNA were cloned with use of In Fusion cloning kit (Takara Bio, USA) according to manufacturer instruction. 5 µL of reaction were taken to transform attached to cloning kit chemocompetent E. Coli which seeded on LB agarose plates with 100 µg/mL of ampicillin (MilliporeSigma, USA). Positive clones identified by colony PCR with primers IRseqF and Neo3R flanking cloning site in vector. Plasmids carrying proper size insert isolated from overnight E. Coli culture by Plasmid Miniprep DNA Purification Kit (EurX, Poland).

### 2.4 Real-time quantitative polymerase chain reaction

Total RNA was extracted using the RNeasy Plus Mini Kit (Qiagen, Hilden, Germany, 74104) following the manufacturer’s instructions. cDNA was synthesized from 2 µg of RNA using the RevertAid First Strand cDNA Synthesis Kit (Thermo Scientific, Karlsruhe, Germany, K1621) also according to the manufacturer’s guidelines. Quantitative PCR was performed in triplicate on a Roche LightCycler 480 system using Power SYBR Green PCR Master Mix (Applied Biosystems, Darmstadt, Germany, 4368702), 300 nM of each primer (**Table 1**) and 1/15 of the cDNA stock. Gene expression levels were calculated using the Pfaffl method (18) and normalized to ACTB expression.

**Table 1:** Primers sequence for qRT-PCR.

Gene	Primer	Sequence
*ACTB*	sense	5’-GCCCTGAGGCACTCTTCCA-3’
	antisense	5’-TTGCGGATGTCCACGTCA-3’
*E-cadherin*	sense	5’-GTCCTGGGCAGACTGAATTT-3’
	antisense	5’- GACCAAGAAATGGATCTGTGG -3’
*ESRP1*	sense	5’-ATAATCAGAGGCACAAACATCACAT-3’
	antisense	5’-ATAATAGAAACTGGGCTACCTCATTGG-3’
*ESRP2*	sense	5’-TGCCACAGAGGATGACTTTG-3’
	antisense	5’-ATTGACTGCTGGGCTCTTTG-3’
*SLUG*	sense	5’-GCCTCCAAAAGCCAAACTACA-3’
	antisense	5’-GAGGATCTCTGGTTGTGGTATGACA-3’
*SNAIL*	sense	5’- TTCTCACTGCCATGGAATTCC-3’
	antisense	5’-GCAGAGGACACAGAACCAGAAA-3’
*TWIST*	sense	5’-GGCCGGAGACCTAGATGTCATT-3’
	antisense	5’-CCACGCCCTGTTTCTTTGAAT-3’
*Vimentin*	sense	5’-CGAGGAGAGCAGGATTTCTC-3’
	antisense	5’- GGTATCAACCAGAGGGAGTGA -3’
*ZEB1*	sense	5’- AAGAATTCACAGTGGAGAGAAGCCA-3’
	antisense	5’- CGTTTCTTGCAGTTTGGGCATT-3’

### 2.5 Western blotting

Cells were lysed in buffer (1 M Tris-HCl pH 6.8, 10% SDS, 50% glycerol) after PBS washes. Equal amounts of protein (10 µg) were separated on 10% SDS-PAGE and transferred to nitrocellulose membranes using the Tetra Cell-Blot system (Bio-Rad) with standard blotting buffer (20 mM Tris, 150 mM glycine, 20% methanol, pH 8.3). Membranes were probed with primary antibodies against E-cadherin (Invitrogen, PAS-32178; 1:1000), ESRP1 (Invitrogen, PA5-115200; 1:1000), ESRP2 (Invitrogen, PA5-113717; 1:1000), Vimentin (Invitrogen, OMA1-06001; 1:3000), ZEB1 (Bethyl, A301921A; 1:1000) and loading control GAPDH (R&D Systems, AF5718; 1:5000), followed by HRP-conjugated secondary antibodies (Jackson ImmunoResearch, West Grove, PA; 1:25,000). Signals were detected using SuperSignal reagents (Thermo Scientific) and visualized on Amersham Hyperfilm ECL (GE Healthcare). Band intensities were quantified with ImageJ and normalized to GAPDH.

### 2.6 Cell scratch wound assay

Cell migration was assessed using a scratch wound healing assay. UM-UC-3 cells were seeded in 6-well plates and cultured until a confluent monolayer was formed. A uniform scratch was created in each well using a sterile 200 µL pipette tip. Detached cells were gently washed away with PBS, and cells were then cultured in serum-reduced medium (1% FBS) to minimize proliferation during the migration period. Wound closure was monitored by phase-contrast microscopy at 0, 24, and 48 hours post-scratch. Images were acquired at defined positions along the wound using the same magnification.

### 2.7 Alamar blue assay

Cells were seeded at a density of 3 × 10^4^ cells per well in a 24-well culture plate. The assay was performed at 24, 48, and 72 hours after seeding. At each time point, cell viability was assessed using a 10% Alamar Blue solution. Briefly, the culture medium was removed and 200 µL of the Alamar Blue solution was added to each well, followed by incubation for 1 hour at 37 °C. After incubation, 100 µL of the supernatant was transferred to a 96-well plate (TPP), and fluorescence was measured using a VICTOR™ Multilabel Plate Reader (PerkinElmer) with a 1-second exposure time.

### 2.8 Spheres forming assay

On the bottom of 12 well culture plate poured 0,5 mL of 1% hot agarose solution in PBS. When solidified seeded 1*10^5^ cells per well in 1 mL of culture medium. Spheroid cultured for 10 days and photographed on reverted microscope (Olympus, Poland). To estimate amount of cell in obtained spheroids, spheres collected and suspended in PBS with 0.1% TritonX-100 and 1 ug/mL Ethidium Bromide to stain DNA.

### 2.9 Colony formation assay

UM-UC-3 cell line stably expressing ESRP1 and ESRP2 were plated at a cell density of 500 cells per well in 6-well culture dishes, and incubated in complete medium for two weeks at 37˚C. The plates were washed and stained with 0.5% crystal violetat room temperature for 15 min. Colonies which consisted of >50 cells were counted under the microscope (Olympus, Poland).

### 2.10 Flow cytometry

Flow cytometric analyses were conducted to evaluate both surface marker expression and cell death. For surface marker detection, UM-UC-3 cells were stained with PE-conjugated mouse anti-human CD133 antibody (clone W6B3C1; BD Pharmingen™, NJ, USA) and PE-conjugated anti-CD44 antibody (Symbios Sp. z o.o., Gdańsk, Poland), following the manufacturers’ protocols. Unstained cells served as negative controls. Samples were analyzed on a FACSAria I flow cytometer (BD Biosciences, Haryana, India), with fluorescence detected in the PE channel. Quantification of CD133^+^ and CD44^+^ populations was based on standard gating strategies.

To assess apoptosis, 1 × 10^6^ cells were washed in 1× binding buffer and stained with FITC-conjugated Annexin V (BD Biosciences) for 15 minutes at room temperature in the dark. After centrifugation, cells were incubated with propidium iodide (PI) for an additional 15 minutes. Samples were analyzed using the same flow cytometer, with fluorescence detected in the FITC and PE channels. Cells were classified as viable, early apoptotic, or late apoptotic/necrotic based on Annexin V and PI staining patterns.

### 2.11 Statistical analysis

Statistical analyses of qRT-PCR data, gene expression, migration rate, and colony formation were performed using GraphPad Prism (version X.X, GraphPad Software, San Diego, CA, USA). Normalized relative expression levels were used to calculate mean values and standard deviation (SD) from at least three independent biological replicates, as represented by columns and error bars in the figures. Data distribution was assessed prior to statistical testing. Statistical significance was evaluated using two-tailed Student’s *t*-test for pairwise comparisons and one-way or two-way ANOVA with Bonferroni’s *post hoc* test for multiple comparisons, as indicated in the figure legends. In all figures, *p*-values are represented as *p* < 0.05; *p < 0.01; **p < 0.001; ***p < 0.0001.

### 2.12 Correlation and survival analysis

Overall patient survival for ESRP1 and ESRP2-high/low bladder urothelial carcinoma tumors of TCGA, PanCancer Atlas were obtained from cBioPortal (19) compared using a log-rank (Mantel-Cox) test. Gene expression data for ESRP1 and ESRP2 were obtained either from cBioPortal (for TCGA) or NCBI GEO database [GEO accession no. GSE128702 (20), GSE57933 (21)].

## 3 Results

### 3.1 ESRP2 expression correlates with luminal phenotype and favorable patient survival

**Figure 1:**
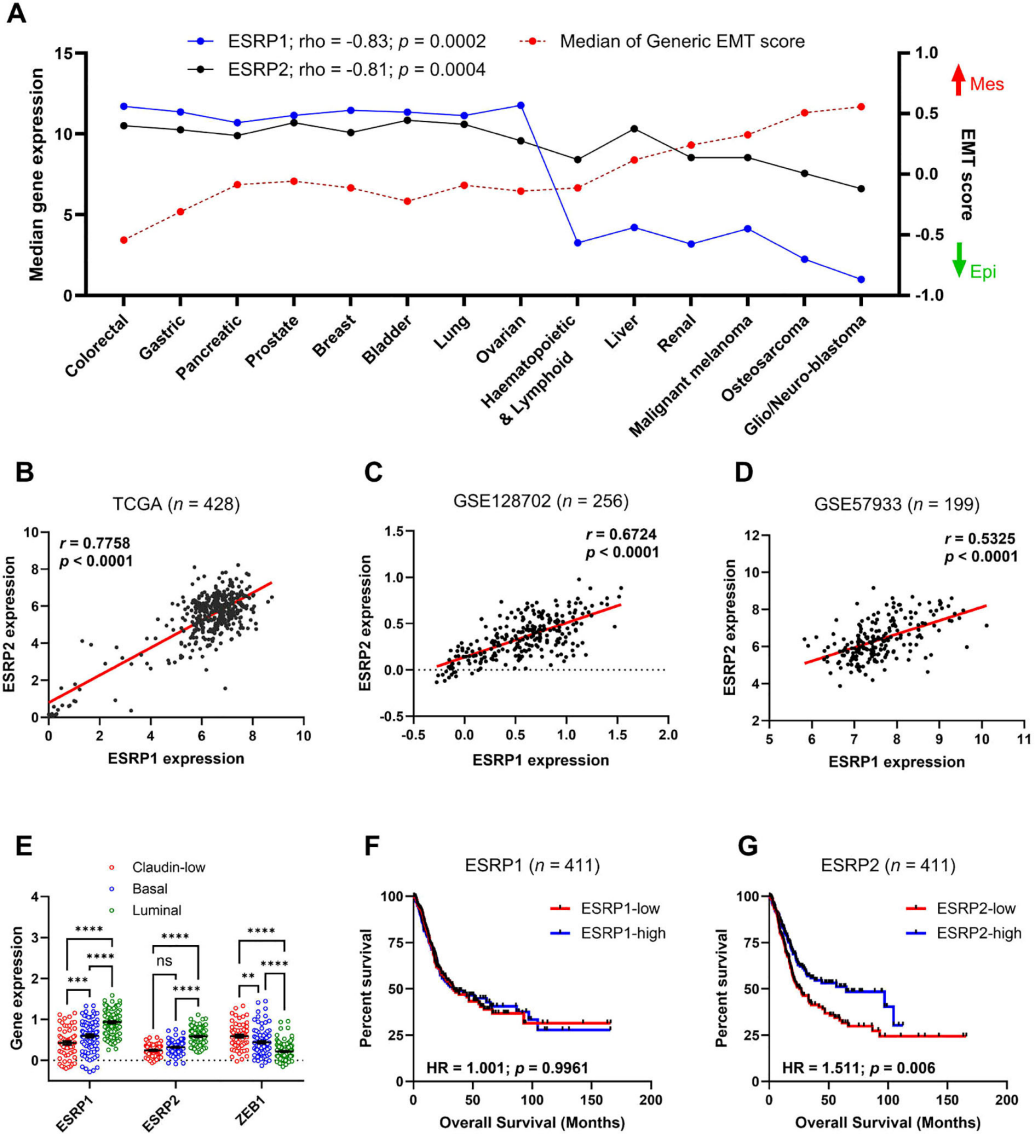
Correlation of ESRP1 and ESRP2 with EMT score, molecular subtype and bladder cancer survival. **(A)** Dot plot showing median ESRP1 (blue) and ESRP2 (black) expressions and median generic EMT score (red) of different cancer types from the Cancer Cell Line Encyclopedia (CCLE). EMT score closer to + 1.0 is more mesenchymal-like (Mes), whereas an EMT score closer to − 1.0 is more epithelial-like (Epi). Pearson correlation coefficients between ESRP1 or ESRP2 expression and EMT score were calculated **(B-D)** Dot plots showing correlation between ESRP1 and ESRP2 expressions in bladder cancer samples in TCGA **(B)**, GSE128702 **(C)** and GSE57993 **(D)**. Correlation between the two variables was evaluated by Pearson’s correlation test. **(E)** Differential expressions of ESRP1, ESRP2, and ZEB1 in molecular subtypes of muscle-invasive bladder cancer (MIBC) derived from GSE87304. **(F, G)** Kaplan Meier survival plots showing overall survival in TCGA bladder cancer patients with ESRP1 **(F)**, ESRP2 **(G)** high patients **(in red)** and ESRP1, ESRP2 low patients (in blue). *** p < 0.001; **** p < 0.0001.

Based on the generic EMT scoring method computed by a previous study (22), we have identified that cancer types of Cancer Cell Line Encyclopedia (CCLE) with lower EMT scores (close to -1.0, epithelial) had higher ESRP1 and ESRP2 expressions, whereas those with higher EMT scores (close to +1.0, mesenchymal) had lower ESRP1 and ESRP2 expressions (**Figure 1A**). Accordingly, strong negative correlations between ESRP1/ESRP2 expression and EMT score (rho= -0.83, -0.81 respectively) were observed, indicating that both proteins are preferentially associated with epithelial phenotypes. Next, we assessed multiple datasets on bladder cancer and identified a strong positive correlation between ESRP1 and ESRP2 expressions (**Figures 1B–D**). To investigate the clinical relevance of ESRP family members in bladder cancer, we analyzed their expression across molecular subtypes of muscle invasive bladder cancer (MIBC) in TCGA dataset. Both ESRP1 and ESRP2 were significantly enriched in luminal tumors with epithelial characteristics and downregulated in claudin-low and basal subtypes exhibiting high EMT activity (**Figure 1E**). In contrast, EMT transcription factor ZEB1 was enriched in claudin-low tumors and reduced in luminal tumors showing an inverse expression pattern relative to ESRP1/2. To evaluate the prognostic impact of ESRP family members, we performed survival analysis on the TCGA cohort. High ESRP2 expression was significantly associated with improved overall survival (HR = 1.511, *p* = 0.006), whereas ESRP1 expression had no significant prognostic value (HR = 1.001, *p* = 0.9961) (**Figures 1F, G**). These findings suggest that ESRP2, but not ESRP1, may serve as a clinically relevant marker associated with epithelial differentiation status and favorable outcome in bladder cancer patients.

### 3.2 EMT status in bladder cancer cells aligns with epithelial marker loss and ZEB1 upregulation

**Figure 2:**
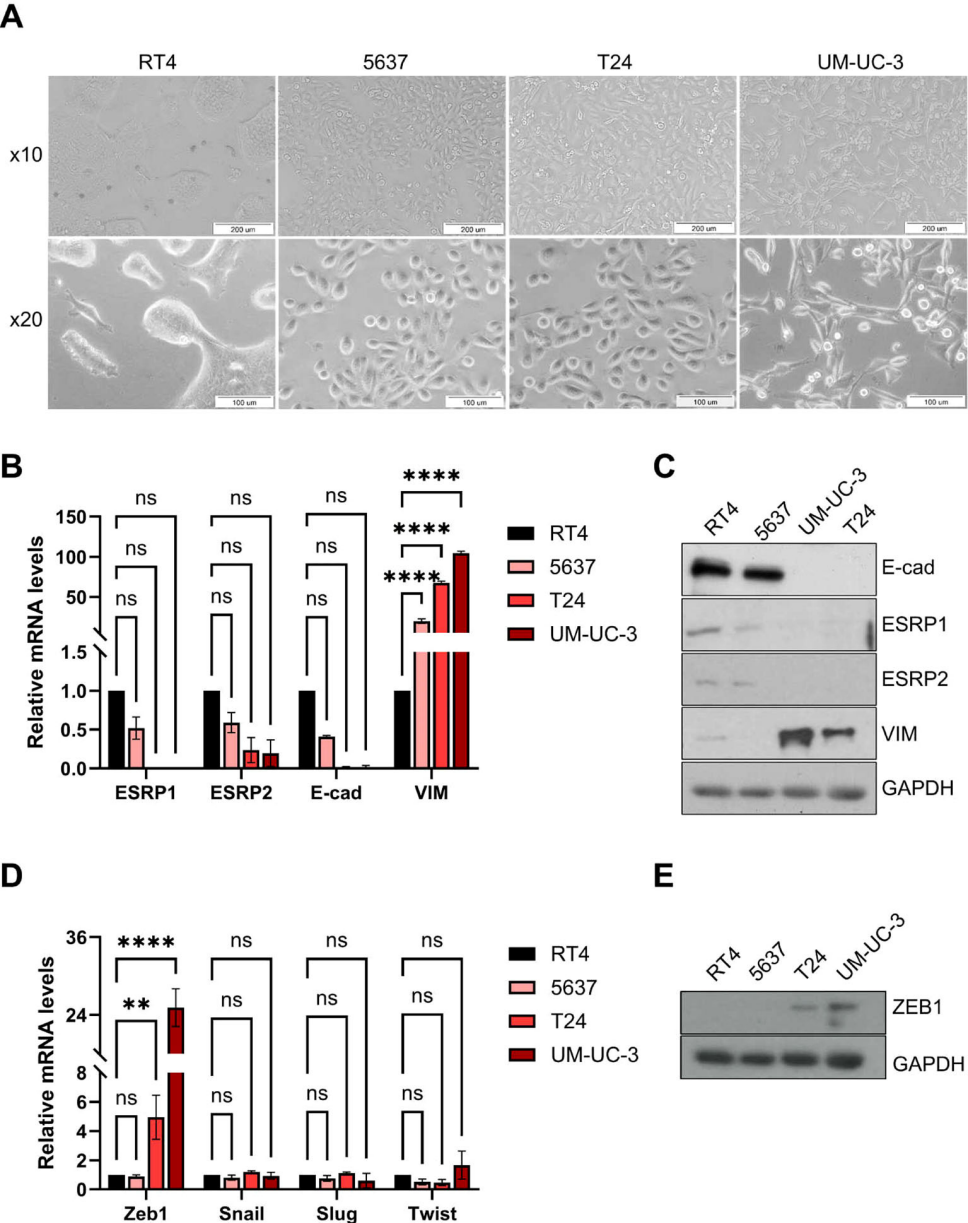
Characterization of bladder cancer cell lines along the EMT spectrum. **(A)** Light microscope photographs of cells showing the morphology of RT-4, 5637, T-24, and UM-UC-3 cell lines. Images were acquired with scale bars of 200 µm (upper panel) and 100 µm (lower panel). **(B, D)** Quantitative RT-PCR analysis of epithelial and mesenchymal markers. Data represent mean ± SD from three independent experiments. **(C, E)** Representative immunoblots showing protein expression levels of indicated markers. ** p < 0.01; **** p < 0.0001.

To characterize the EMT landscape in bladder cancer, we analyzed four commonly used urothelial carcinoma cell lines representing different levels of differentiation and invasiveness. Phase-contrast microscopy revealed distinct morphological features that aligned with their phenotypic states (**Figure 2A**). RT-4 cells exhibited a typical cobblestone-like appearance, characteristic of well-differentiated epithelial cells, whereas 5637 cells showed a mixed morphology with epithelial islands and scattered spindle-shaped cells, consistent with an intermediate EMT state. In contrast, T24 and UM-UC-3 cells displayed elongated, fibroblast-like shapes indicative of mesenchymal morphology and a more aggressive phenotype. Gene and protein expression analyses through real time-quantitative PCR (RT-qPCR) showed that RT-4 cells exhibited expression of epithelial markers (ESRP1, ESRP2, E-cadherin) together with low Vimentin levels, consistent with an epithelial phenotype (**Figures 2B, C**). In contrast, T24 and UM-UC-3 cells showed strong downregulation of epithelial markers and upregulation of Vimentin, indicative of a mesenchymal state. The intermediate profile of 5637 cells, characterized by co-expression of epithelial and mesenchymal markers, suggested a hybrid phenotype. These transcriptomic patterns were largely reflected at the protein level by Western blot analysis, although partial discrepancies between mRNA and protein abundance were observed for selected EMT markers, particularly in the intermediate 5637 cell line. Such partial discordance between transcript and protein levels is consistent with hybrid EMT states and may reflect post-transcriptional regulation, translational control, or differences in protein stability. To further characterize EMT-associated transcriptional regulators, we analyzed the expression of major EMT transcription factors ZEB1, Snail, Slug, and Twist in bladder cancer cell lines (**Figures 2D, E**). ZEB1 showed the most pronounced upregulation at both the mRNA and protein levels, particularly in UM-UC-3 cells, with lower expression in T24 and minimal levels in RT-4 and 5637. Snail, Slug, and Twist were expressed at relatively low levels across all lines, with only modest increases in mesenchymal-like cells. These findings indicate that ZEB1 is the most prominently expressed EMT-associated transcription factor in this model. Together, these results support the classification of the bladder cancer cell lines along the EMT spectrum. The observed gene and protein expressions align with their morphological characteristics and suggest an association with their differentiation status and invasive potential.

### 3.3 ESRP2 silencing induces EMT-associated transcriptional changes in epithelial bladder cancer cells

**Figure 3:**
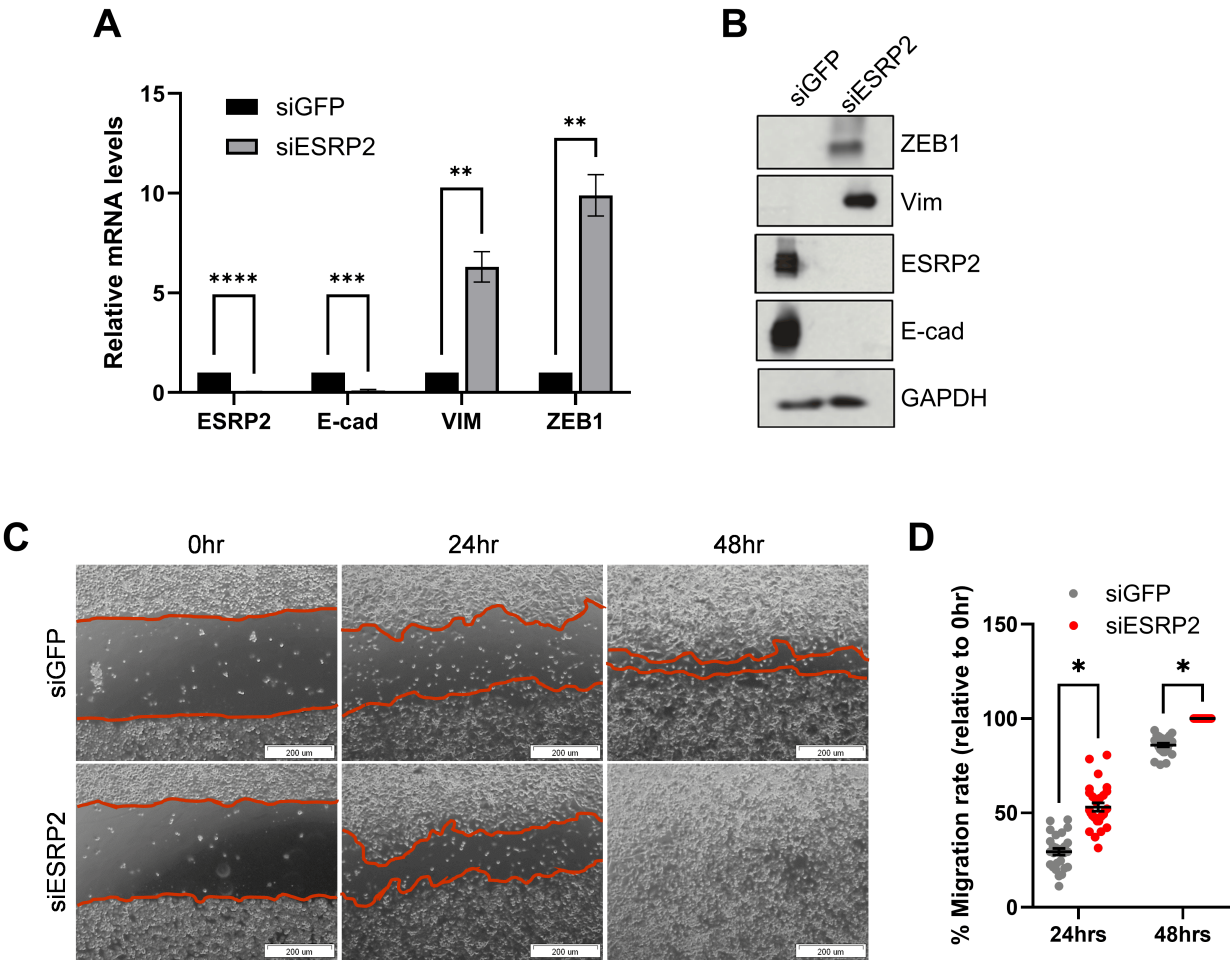
ESRP2 silencing promotes EMT-like changes in RT4 cells. **(A)** qRT-PCR analysis of *ESRP2*, *E-cad*, *Vim* and *ZEB1* mRNA levels after siESRP2 or siGFP transfection. Data are mean ± SD (n = 3); **p < 0.01, ***p < 0.001, ****p < 0.0001. **(B)** Western blot confirming ESRP2 knockdown and showing decreased E-cadherin, increased Vimentin and ZEB1 protein levels. **(C)** Representative phase-contrast images from scratch wound assay at 0, 24, and 48 hours post-scratch showing accelerated wound closure in siESRP2-transfected cells. Scale bar = 200 µm. **(D)** Quantification of wound closure showing significantly increased migration rate in ESRP2-silenced cells compared with controls (*p < 0.05, two-tailed Student’s t-test*). * p < 0.05.

To explore the functional role of ESRP2 in maintaining epithelial identity, we performed siRNA-mediated knockdown in RT-4 cells, a well-differentiated epithelial bladder cancer model. Efficient silencing of ESRP2 was confirmed by qRT-PCR and western blotting (**Figures 3A, B**). Loss of ESRP2 was accompanied by downregulation of E-cadherin and increased expression of Vimentin and ZEB1, consistent with EMT-associated marker changes indicative of a partial EMT state.

To assess whether these molecular alterations translate into functional changes, we evaluated cell motility using a scratch wound assay. ESRP2-depleted RT-4 cells exhibited accelerated wound closure compared with siGFP-transfected controls, indicating enhanced migratory capacity (**Figures 3C, D**). Similar effects were observed upon ESRP1 knockdown (**Supplementary Figure 1**), supporting a shared role of both splicing regulators in maintaining epithelial-associated gene expression patterns and limiting mesenchymal and migratory phenotypes.

### 3.4 ESRP2 re-expression promotes epithelial-like features and suppresses migration in mesenchymal bladder cancer cells

**Figure 4:**
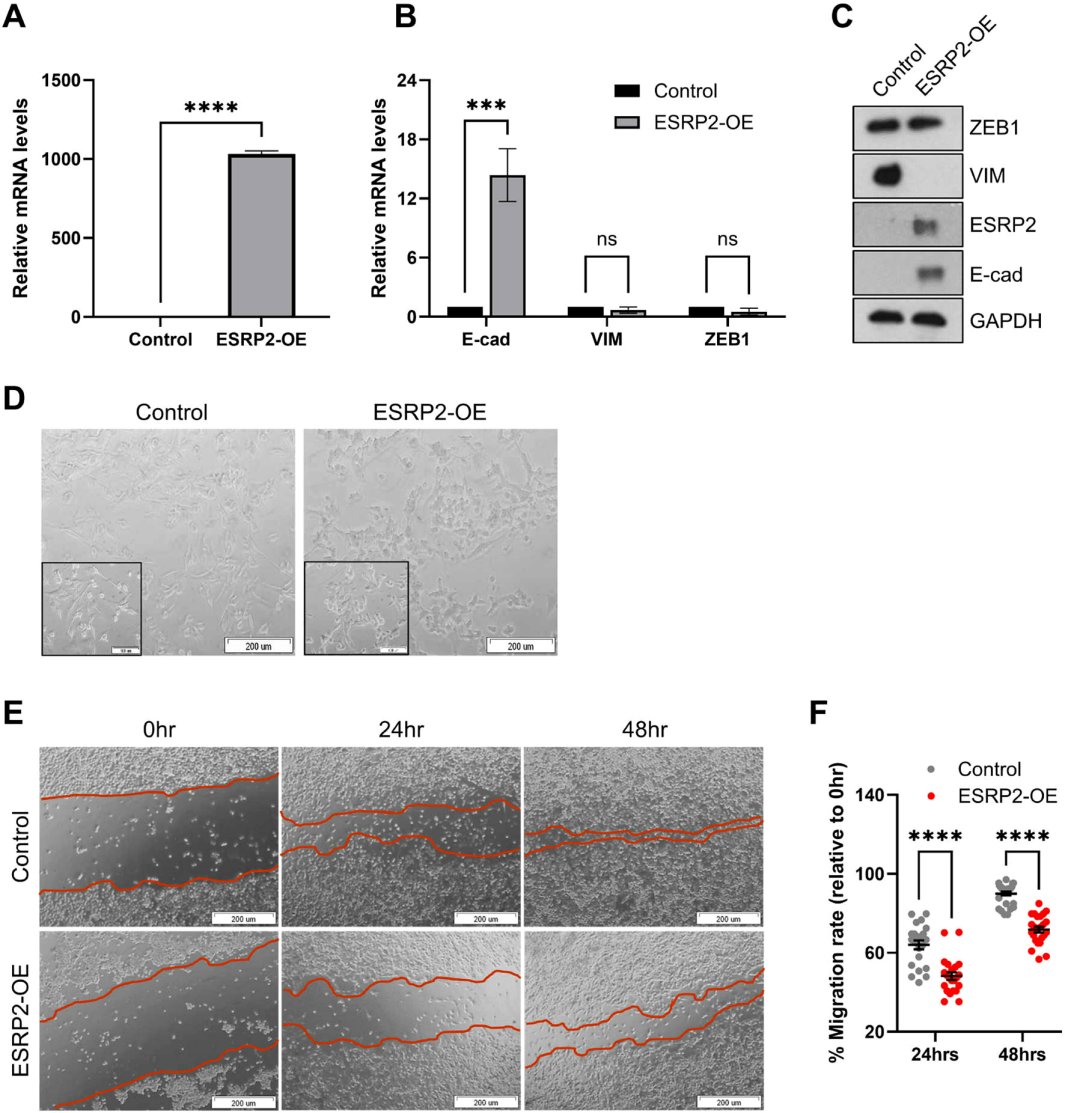
ESRP2 overexpression leads to morphological and molecular changes in UM-UC-3 cells. **(A–C)** qRT-PCR and representative Western blot analysis of ESRP2, E-cadherin (E-cad), vimentin (VIM), and ZEB1 expression following ESRP2 overexpression in UM-UC-3 cells. Quantitative data represent mean ± SD from three independent experiments. **(D)** Representative microscopic images of UM-UC-3 cells overexpressing ESRP2 or transduced with empty vector (control). Scale bar = 200 μm. **(E)** Representative images and **(F)** quantification of in vitro scratch wound healing assays performed on control and ESRP2-overexpressing UM-UC-3 cells. Data represent mean ± SD from three independent experiments. Statistical significance was assessed using a two-tailed Student's t-test (*p < 0.05; **p < 0.01; ***p < 0.001; ****p < 0.0001).

To determine whether ESRP2 re-expression is associated with epithelial-like changes in mesenchymal bladder cancer cells, we overexpressed ESRP2 in UM-UC-3 cells. This was accompanied by increased E-cadherin expression and reduced Vimentin and ZEB1 expression at both mRNA and protein levels (**Figures 4A–C**). Morphologically, ESRP2-overexpressing cells exhibited a shift from spindle-shaped to cobblestone-like appearance (**Figure 4D**). Functionally, ESRP2 significantly impaired wound closure in scratch assay, indicating reduced migratory capacity (**Figures 4E, F**). Also, overexpression of ESRP1 induced similar but less pronounced molecular and phenotypic effects (**Supplementary Figure 2**).

### 3.5 ESRP2 impairs clonogenic growth without inducing apoptosis

**Figure 5:**
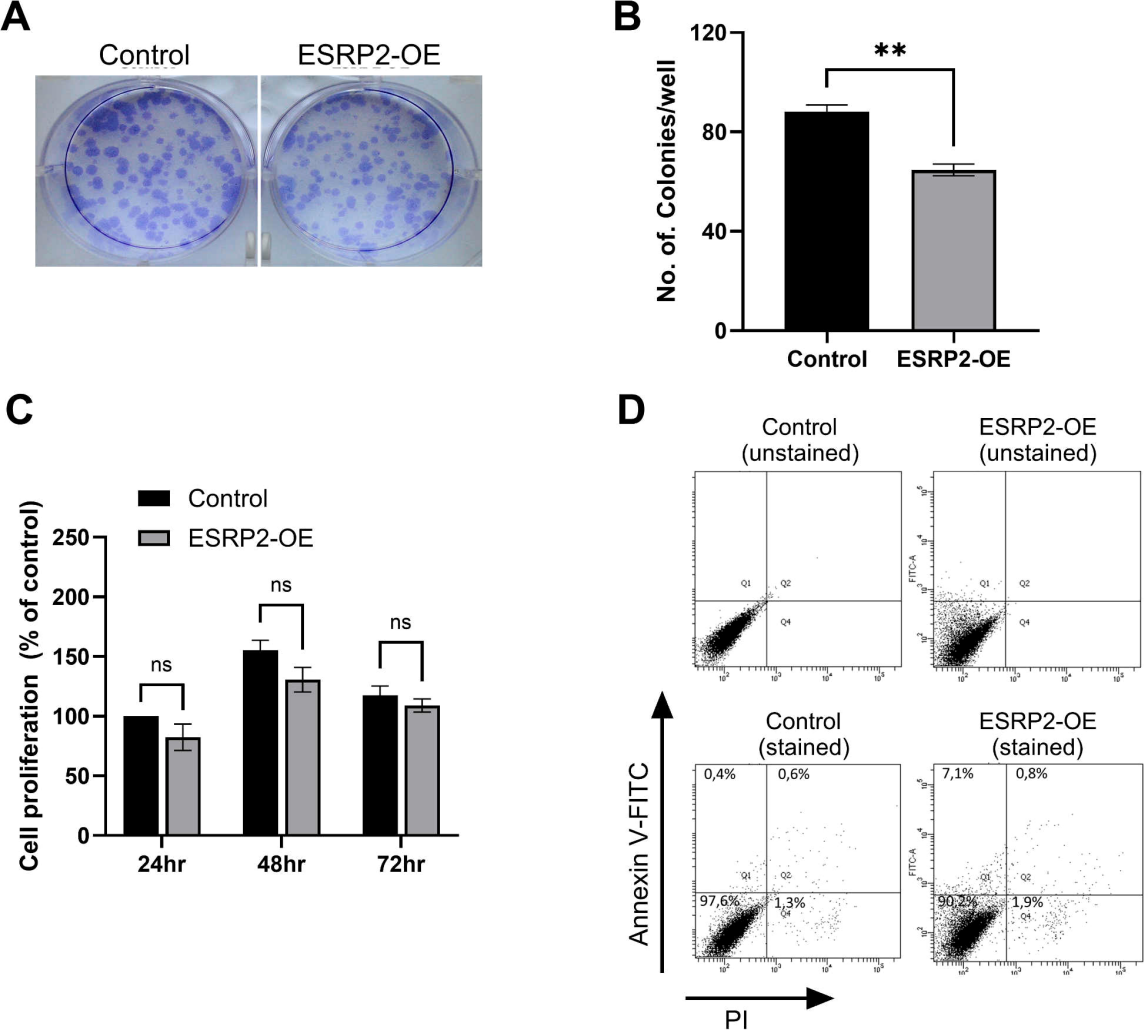
ESRP2 overexpression suppresses clonogenic growth without inducing apoptosis. **(A)** Representative images of the colony formation potential of UM-UC-3 cells transduced with GFP (control), and ESRP2. **(B)** Quantification of colony numbers. Data represent mean ± SD from three independent experiments. Statistical significance was assessed using a two-tailed Student’s *t*-test. **(C)** Cell proliferation assessed by Alamar Blue^®^ assay in UM-UC-3 cells overexpressing ESRP2. Data are shown as percentage change relative to control cells at 24 h (set as 100%) and represent mean ± SD from three independent experiments; no statistically significant differences were observed. **(D)** Representative Annexin V-FITC/PI dot plots showing apoptosis in control, and ESRP2-overexpressing cells. ** p < 0.01.

Next, we assessed the tumorigenic potential following ESRP2 overexpression. ESRP2 significantly reduced colony number compared to control cells, while short-term proliferation assessed by Alamar Blue assay showed a non-significant downward trend (**Figures 5A–C**). Furthermore, flow cytometric analysis after Annexin V/PI staining revealed no increase in apoptotic populations (**Figure 5D**), indicating that the observed reduction in clonogenic growth is not attributable to increased cell death. In contrast, ESRP1 overexpression had no significant effect on clonogenicity or apoptosis (**Supplementary Figure 3**).

### 3.6 ESRP2 impairs anchorage-independent growth and alters the composition of stem-like subpopulations

**Figure 6:**
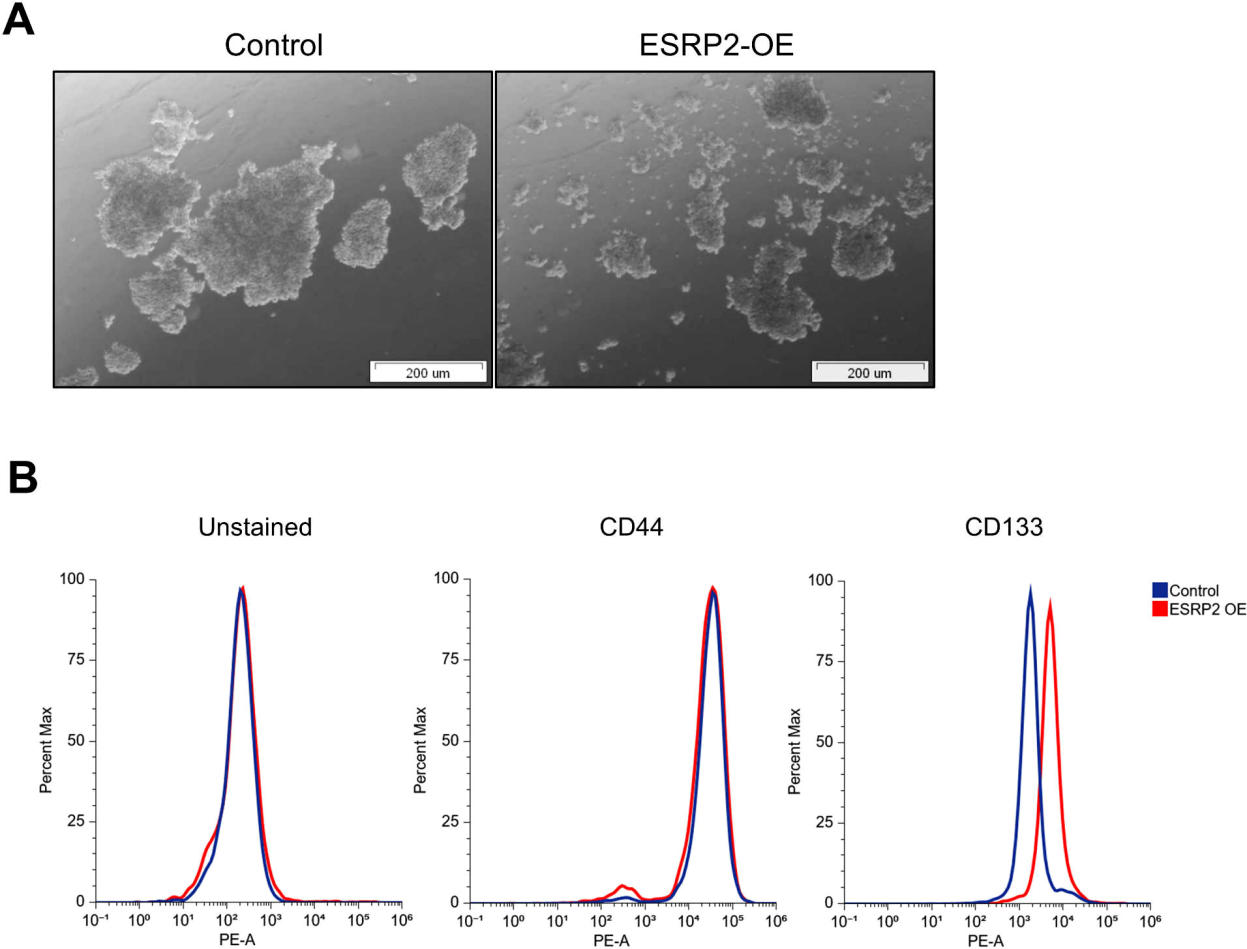
ESRP2 overexpression limits spheroid formation and alters the composition of stem-like subpopulations. **(A)** Representative brightfield images of spheroids formed by UM-UC-3 cells expressing GFP (Control) and ESRP2 under low-attachment 3D culture conditions. Cells were seeded at 2 × 10^5^ cells/well and cultured for 3 days. Scale bar = 200µm. **(B)** Flow cytometric analysis of CD133 and CD44 surface marker expression in control and ESRP2-overexpressing UM-UC-3 cells. Histograms show PE fluorescence intensity compared to unstained controls. Data represent mean ± SD from three independent experiments. Statistical significance was assessed using a two-tailed Student’s *t*-test.

To assess anchorage-independent growth, UM-UC-3 cells were cultured under low-attachment conditions. Control cells formed large, compact spheroids, whereas ESRP2-overexpressing cells generated smaller and more disorganized structures, indicating impaired self-assembly and reduced 3D growth potential (**Figure 6A**). ESRP1-overexpressing cells also exhibited looser spheroid architecture but showed no consistent reduction in spheroid number or size (**Supplementary Figure 4A**).

Flow cytometric analysis revealed that ESRP2 overexpression did not affect the proportion of CD44^+^ cells but was accompanied by a modest increase in the CD133^+^ population (**Figure 6B**). ESRP1 overexpression did not significantly alter the expression of either marker (**Supplementary Figure 4B**).

These findings indicate that the impaired spheroid formation observed upon ESRP2 overexpression is not associated with a depletion of canonical cancer stem cell surface markers. Instead, ESRP2 may shift the balance between distinct stem-like subpopulations, by altering their relative representation as defined by CD133 and CD44 expression. This suggests that ESRP2 modulates cancer cell plasticity not by eliminating stemness, but by altering its phenotypic composition.

**Figure 7:**
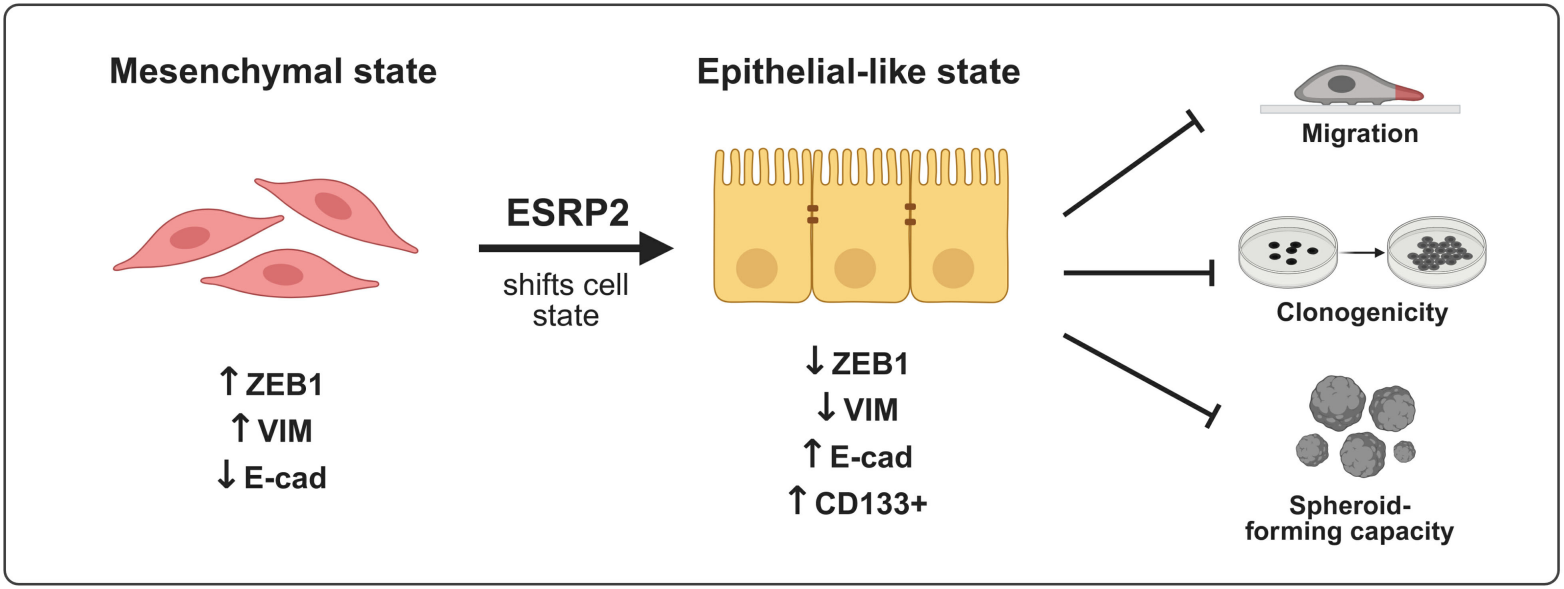
Proposed model of ESRP2-mediated regulation of cell-state identity in bladder cancer. ESRP2 constrains EMT-associated cellular programs linked to ZEB1 expression, thereby limiting tumor cell plasticity and promoting epithelial-associated features. This is accompanied by reduced migratory and clonogenic capacity, restoration of epithelial morphology and E-cadherin expression, and a qualitative reorganization of marker-defined stem-like subpopulations without global suppression of stemness.

Together, these findings indicate that ESRP2 does not induce a complete mesenchymal-to-epithelial transition but rather shifts bladder cancer cells toward epithelial-associated features. As summarized in **Figure 7**, ESRP2 restoration is associated with reduced ZEB1 expression, decreased Vimentin levels and migratory capacity, and re-establishment of epithelial morphology and E-cadherin expression. In parallel, ESRP2 does not eliminate stem-like subpopulations but alters their marker-defined composition, including a modest increase in CD133^+^ cells without affecting CD44 expression. Collectively, these coordinated effects support a model in which ESRP2 functions as a post-transcriptional regulator that constrains EMT-associated programs linked to ZEB1 expression, thereby limiting tumor cell plasticity and supporting epithelial lineage identity.

## 4 Discussion

Epithelial-to-mesenchymal transition (EMT) plays a central role in bladder cancer (BC) progression, metastasis, and therapeutic resistance (23, 24). While EMT is traditionally associated with transcriptional repressors such as ZEB1, Snail, and Twist (25, 26), accumulating evidence highlights the importance of post-transcriptional mechanisms—particularly alternative splicing—in modulating epithelial plasticity (27, 28). Notably, EMT-inducing signals such as TGF-β can downregulate ESRPs through transcriptional repression by δEF1/ZEB1, linking canonical EMT pathways with alternative splicing regulation in multiple epithelial contexts (29).

In this study, we identify ESRP2 as a clinically and functionally relevant regulator of epithelial identity in BC. Transcriptomic analyses revealed that high ESRP2 expression correlates with improved overall survival, especially in luminal tumor subtypes, supporting its utility as a prognostic biomarker. Functional studies further confirmed that ESRP2 is preferentially expressed in epithelial-like BC cell lines and downregulated in mesenchymal counterparts, consistent with an association with epithelial differentiation and reduced aggressiveness.

Loss-of-function studies showed that ESRP2 knockdown in epithelial BC cells was associated with EMT-associated transcriptional changes, including E-cadherin downregulation and increased expression of Vimentin and ZEB1, hallmarks of partial EMT.

In contrast, gain-of-function studies demonstrated that ESRP2 re-expression in mesenchymal cells restored epithelial morphology, impaired 3D spheroid formation, and showed a trend toward reduced proliferation (30). Interestingly, these phenotypic effects were not accompanied by a decrease in the CD44^+^ cell population. Instead, flow cytometry revealed a modest increase in CD133^+^ cells following ESRP2 overexpression.

These observations suggest that ESRP2 does not globally suppress stem-like traits, but rather alters the composition of stem-like subpopulations. Specifically, ESRP2 altered the relative representation of marker-defined stem-like subpopulations, driven by changes in the CD133^+^ population, while the proportion of CD44^+^ cells remained unchanged (30). The lack of change in CD44 expression, despite a clear suppression of migratory capacity and clonogenic growth, further supports the hypothesis that ESRP2 acts through isoform switching or non-canonical mechanisms, rather than by directly repressing classical surface markers (10, 13, 14, 30). The divergent behavior of stemness-associated markers strengthens this interpretation: while CD44^+^ populations—commonly linked to EMT and mesenchymal stemness—remained stable (14), ESRP2 overexpression led to a modest expansion of the CD133^+^ subpopulation (30). This shift may reflect partial reprogramming of the cancer stem cell compartment toward epithelial-associated states without eliminating stem-like properties (4, 6, 30). The concurrent impairment in spheroid formation and anchorage-independent growth suggests that ESRP2 affects 3D self-organization and tumor cell plasticity, rather than directly suppressing functional tumor-initiating capacity.

Although ESRP1 shares structural similarity with ESRP2 and partially overlaps in function (13, 16, 31), our comparative analysis indicates that ESRP2 exerts a broader and more robust impact on oncogenic traits in BC. Previous studies have shown that ESRP1 can suppress invasiveness in epithelial cancers, such as lung adenocarcinoma (32), suggesting conserved tumor-suppressive roles among ESRP paralogs. While both ESRPs affect EMT markers, only ESRP2 significantly impaired clonogenic growth and 3D architecture. Importantly, despite partially overlapping *in vitro* effects, only ESRP2 showed a significant association with patient survival in TCGA bladder cancer cohorts. This discrepancy suggests that ESRP2-dependent splicing programs may be more broadly engaged or more stable at the tumor level, whereas ESRP1-related effects may be context-restricted or compensated *in vivo*. Moreover, ESRP2 expression displayed greater dynamic range across molecular subtypes, potentially enhancing its prognostic relevance. These distinctions highlight a more pronounced role for ESRP2 in maintaining epithelial homeostasis. The functional divergence between ESRP1 and ESRP2 may arise from differences in splicing target specificity, localization, or interaction partners, underscoring the importance of studying these factors independently. This is supported by evidence that ESRP1 and ESRP2 control cell motility via distinct mechanisms (33). Moreover, ESRPs may exert context-dependent roles, as highlighted by their proposed oncogenic potential in aggressive prostate cancer (34).

Notably, ESRP2-induced growth suppression was not associated with increased apoptosis, as verified by Annexin V/PI staining, suggesting an association with epithelial differentiation and reduced proliferative activity rather than cytotoxic effects. In hepatocellular carcinoma, ESRP2 loss has been shown to activate pro-oncogenic TAK1 signaling via alternative splicing, reinforcing its broader tumor-suppressive role (35). Further studies are needed to identify ESRP2-dependent splicing targets in bladder cancer. Mechanistically, ESRP2 may exert its tumor-suppressive effects through alternative splicing of genes involved in adhesion, signaling, and plasticity. Known ESRP targets include CD44, FGFR2, and ENAH—all linked to EMT and stemness (13, 16). The isoform balance between CD44 variant (CD44v) and standard (CD44s) isoforms is particularly relevant: CD44v supports epithelial identity, while CD44s promotes invasiveness (14, 36). While this mechanism was not directly investigated in the present study, evidence indicates that CD44s sustains EMT through a ZEB1 feedback loop, reinforcing stem-like traits (37). Previous studies have shown that ESRP1/2 promote variable exon inclusion, favoring CD44v expression, whereas ESRP loss drives a switch toward CD44s expression (38). Although our current study did not include isoform-level analysis, it is plausible that ESRP2 constrains EMT-associated plasticity through splicing-mediated mechanisms (39). Therefore, the present study focuses on phenotypic and transcriptional consequences of ESRP2 modulation rather than direct identification of splicing events. Future transcriptome-wide analyses will be instrumental in defining the ESRP2-dependent splicing landscape in bladder cancer. Together, these mechanistic insights provide a foundation for exploring the clinical implications of ESRP2 regulation in bladder cancer.

From a translational perspective, the strong association between high ESRP2 expression and favorable patient outcome, together with its ability to promote epithelial-associated features in mesenchymal-like cells, positions ESRP2 as a promising biomarker and therapeutic target (40, 41). Restoring ESRP2 function or modulating its splicing network may limit tumor plasticity and overcome treatment resistance. Indeed, therapeutic targeting of splicing regulators has gained traction: antisense oligonucleotides, small molecules, and CRISPR-based tools have shown efficacy in redirecting splicing (42–45). The SF3B1 modulator H3B-8800, for example, completed a Phase I trial in myeloid neoplasms, demonstrating on-target effects and favorable safety, including transfusion independence in some patients (46). Likewise, splice-switching oligonucleotides have yielded promising preclinical results in solid tumors by restoring tumor-suppressive isoforms (47). These approaches support the feasibility of targeting splicing in cancers with high plasticity. Additionally, ESRPs have been implicated in hormone signaling in luminal breast cancer, suggesting potential interaction with other lineage-specific pathways (48).

Nevertheless, our study has limitations. All experiments were conducted *in vitro* using established cell lines, which may not fully recapitulate the complexity of the tumor microenvironment. In particular, interactions with stromal, immune, and extracellular matrix components may modulate ESRP2-dependent epithelial–mesenchymal plasticity *in vivo*. *In vivo* studies and patient-derived models are therefore necessary to validate ESRP2’s clinical relevance in bladder cancer. Furthermore, although we characterized the phenotypic consequences of ESRP2 modulation, we did not identify its direct splicing targets, representing an important direction for future investigation.

## 5 Conclusion

Together, our findings support a model in which ESRP2 functions as a post-transcriptional regulator that constrains EMT-associated cellular programs linked to ZEB1 expression, thereby stabilizing epithelial identity and limiting tumor cell plasticity in bladder cancer. Rather than inducing a full mesenchymal-to-epithelial transition, ESRP2 promotes epithelial-associated features, including restoration of epithelial morphology, re-expression of E-cadherin, and reduction of migratory and clonogenic capacity. Importantly, ESRP2 does not abolish stem-like properties but alters the composition of marker-defined stem-like subpopulations, reflecting a qualitative remodeling of cancer cell plasticity rather than global stemness suppression. Collectively, these results position ESRP2 as a clinically relevant functional regulator and prognostic marker associated with epithelial differentiation, providing a conceptual framework for targeting splicing networks to limit tumor adaptability and potentially overcome therapeutic resistance in aggressive bladder cancer.

## Data Availability

The original contributions presented in the study are included in the article/**Supplementary Material**. Further inquiries can be directed to the corresponding author.
