# Supplementary material for: ESRP2 constrains EMT plasticity associated with ZEB1 expression in bladder cancer
Source: Front Oncol. 2026 Mar 30;16:1764850. doi: 10.3389/fonc.2026.1764850 (PMC13070961; doi:10.3389/fonc.2026.1764850)
Supplement: Supplementary file 1 [file DataSheet1.docx]

Supplementary Material

# Supplementary Data


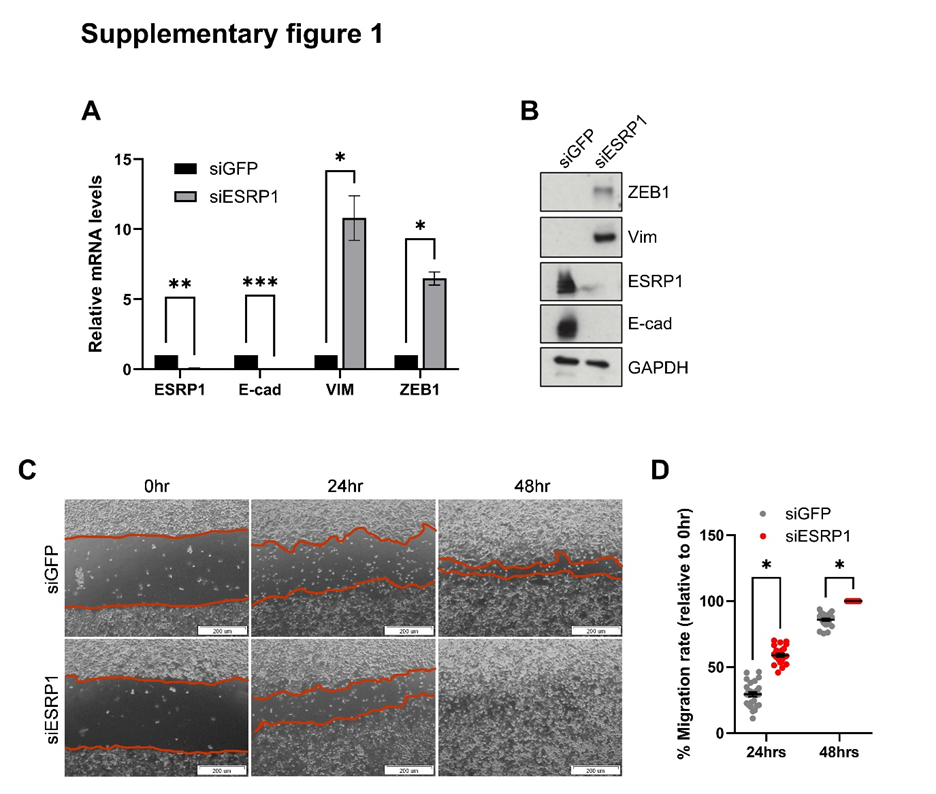


**Supplementary figure 1. ESRP1 silencing promotes EMT-like changes in RT4 cells.**(A) qRT-PCR analysis of *ESRP1*, *E-cad*, *Vim* and *ZEB1* mRNA levels after siESRP1 or siGFP transfection. Data are mean ± SD (n = 3); **p < 0.01, ***p < 0.001, ****p < 0.0001. (B) Representative Western blot confirming ESRP1 knockdown and showing decreased E-cadherin, increased Vimentin and ZEB1 protein levels. (C) Representative phase-contrast images from scratch wound assay at 0, 24, and 48 hours post-scratch showing accelerated wound closure in siESRP1-transfected cells. Scale bar = 200 µm. (D) Quantification of wound closure showing significantly increased migration rate in ESRP1-silenced cells compared with controls. Data represent mean ± SD from three independent experiments. Statistical significance was assessed using a two-tailed Student’s *t*-test (*p* < 0.05).

**
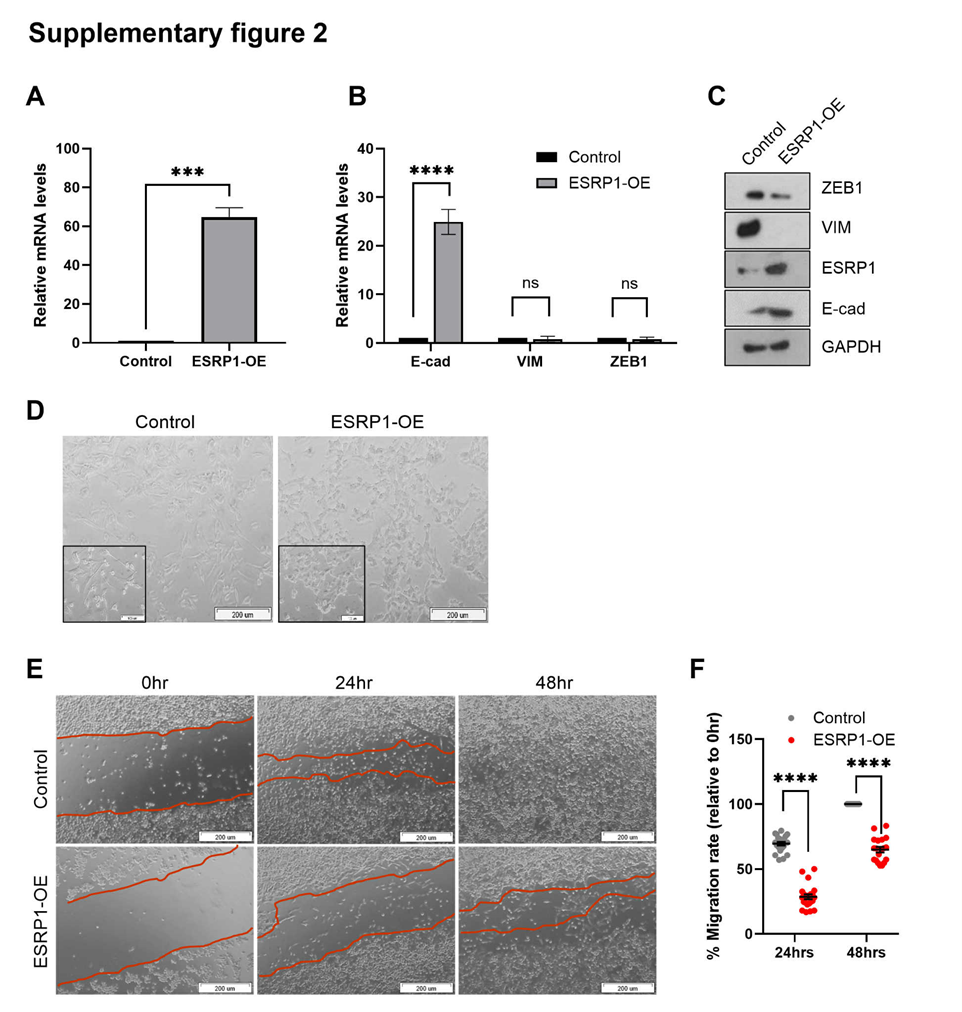
**

**Supplementary figure 2. ESRP1 overexpression leads to morphological and molecular changes of UM-UC-3 cells.** (A,B,C) qRT-PCR and representative Western blot analysis of ESRP1, E-cad, Vim and ZEB1 expression following ESRP1 overexpression in the UM-UC-3 cells. Quantitative data represent mean ± SD from three independent experiments. (D) Representative phase-contrast images showing morphological changes in UM-UC-3 cells overexpressing ESRP1 or transduced with empty vector. Scale bar = 200 µm . (E,F) Representative images and quantification of in vitro scratch wound healing assays performed on ESRP1-overexpressing UM-UC-3 cells. Data represent mean ± SD from three independent experiments. Statistical significance was assessed using a two-tailed Student’s *t*-test.

**
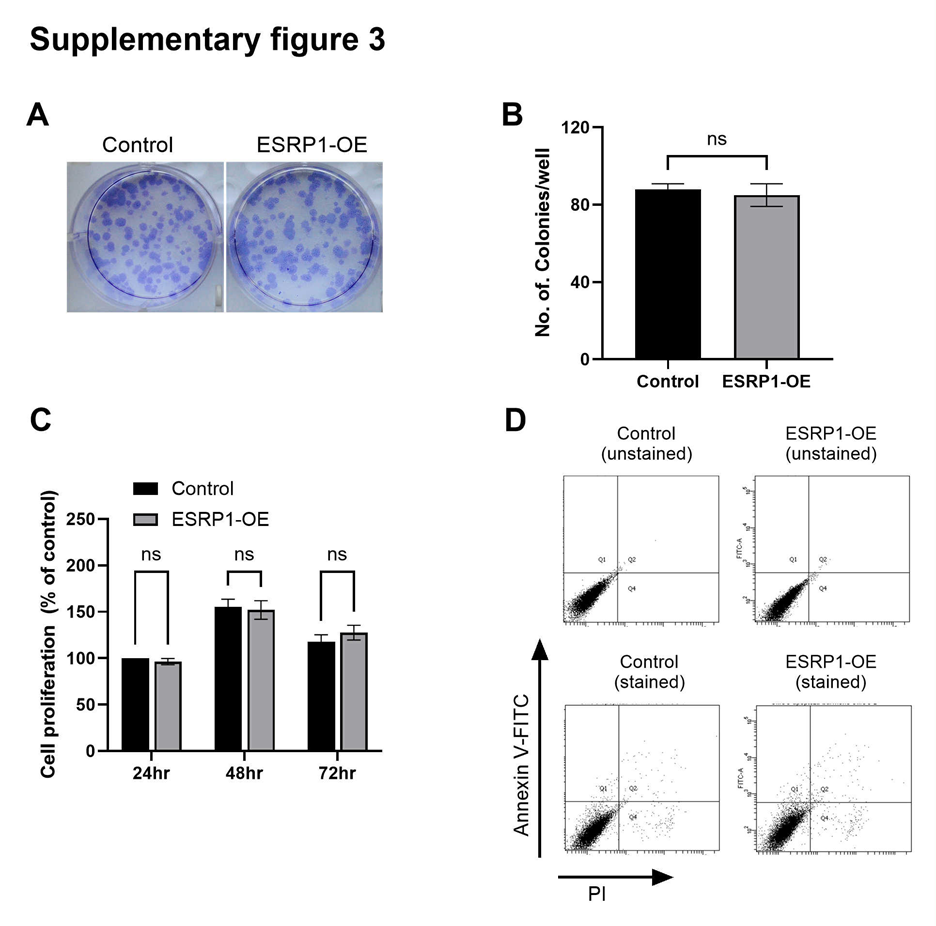
**

**Supplementary figure 3. No significant effect of ESRP1 overexpression on cell growth or apoptosis.** (A) Representative images of the colony formation potential of UM-UC-3 cells transduced with GFP (control), and ESRP1. (B) Quantification of colony numbers. Data represent mean ± SD from three independent experiments; no statistically significant differences were observed. (C) Cell proliferation assessed by Alamar Blue® assay in UM-UC-3 cells overexpressing ESRP1. Data are shown as percentage change relative to control cells at 24 h (set as 100%) and represent mean ± SD from three independent experiments; no statistically significant differences were observed. (D) Representative Annexin V-FITC/PI dot plots showing apoptosis in control and ESRP1-overexpressing cells.

**
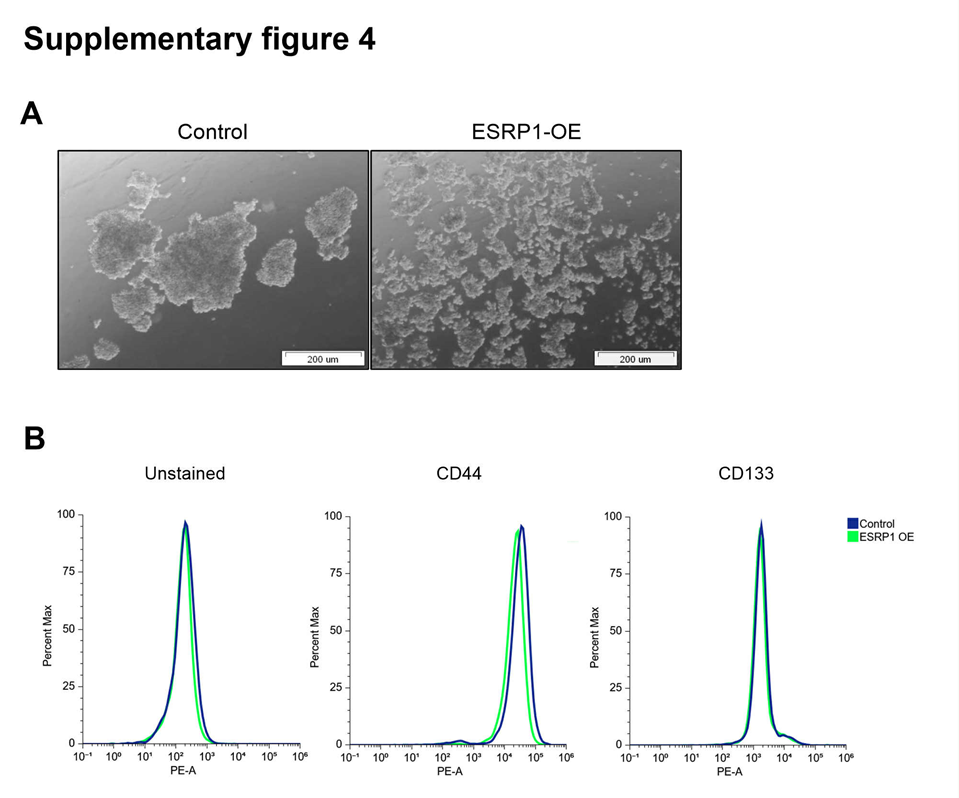
**

**Supplementary figure 4. ESRP1 overexpression alters spheroid morphology without affecting CD44⁺ or CD133⁺ stem-like subpopulations.** (A) Representative brightfield images of spheroids formed by UM-UC-3 cells expressing GFP (Control) and ESRP1 under low-attachment 3D culture conditions. Cells were seeded at 2 × 10⁵ cells/well and cultured for 3 days. Scale bar = 200 µm. (B) Flow cytometric analysis of CD133 and CD44 surface marker expression in control and ESRP1-overexpressing UM-UC-3 cells. Histograms show PE fluorescence intensity compared to unstained controls. Data represent mean ± SD from three independent experiments; no statistically significant differences were observed (two-tailed Student’s *t*-test).
